# Analysis of lymph node metastasis in early gastric cancer: a single institutional experience from China

**DOI:** 10.1186/s12957-020-01834-7

**Published:** 2020-03-20

**Authors:** Jinggui Chen, Guangfa Zhao, Yanong Wang

**Affiliations:** 1 Department of Gastric Surgery, Fudan University Shanghai Cancer Center, No. 270 Dongan Road, Shanghai, 200032 People’s Republic of China; 2 Department of Oncology, Shanghai Medical College, Fudan University, No. 270 Dongan Road, Shanghai, 200032 People’s Republic of China

**Keywords:** Early gastric cancer, Lymph node metastasis, Risk factors

## Abstract

**Background:**

Lymph node metastasis (LNM) has a strong influence on the prognosis of patients with early gastric cancer (EGC). The aim of this study was to reveal the incidence of LNM and evaluate risk factors for LNM to determine the appropriate treatment for EGC in a Chinese population.

**Methods:**

Patients who underwent radical gastrectomy with lymph node dissection for EGC between 2012 and 2017 were retrospectively analyzed. Univariate and multivariate analyses were conducted to identify clinicopathological features that were risk factors for LNM.

**Results:**

A total of 1033 patients with EGC were enrolled. Of these patients, 668 (64.7%) were men, and 365 (35.3%) were women, ranging in age from 19 to 82 years (mean 56.9 ± 10.9 years). LNM was detected in 173(16.7%) patients with EGC. Among 508 patients with mucosal cancer, 44 (8.7%) patients had LNM. In 525 patients with submucosal cancer, the incidence of LNM was 24.6% (129/525). The age, gender, tumor size, type of differentiation, Lauren classification, and lymphovascular and perineural invasion showed a significant correlation with the rate of LNM in EGC by univariate and multivariate analyses. Patients with submucosal gastric cancer had an older age, a higher proportion of proximal lesion, larger tumor size, more frequent lymphovascular invasion, perineural invasion, and more LNM than patients with mucosal gastric cancer.

**Conclusions:**

Our study revealed a relatively high incidence of LNM in EGC, compared with Japanese and Korean cohorts. Female sex, large tumor size, undifferentiated-type, and lymphovascular invasion were independent risk factors for LNM in EGC. Radical gastrectomy with lymphadenectomy should be performed in EGC patients with a high risk of LNM.

## Introduction

Early gastric cancer (EGC), which is defined as tumor confined to the mucosa or submucosa, regardless of the presence or absence of lymph node metastasis (LNM), has a better prognosis than advanced gastric cancer [1]. LNM is deemed as the most important prognostic factor [2, 3]. Previous study reported that the 5-year survival rate was 87.3% in EGC patients with LNM and 94.2% in patients without LNM, respectively [4]. The incidence of LNM in EGC varies obviously in EGC patients with different depth of invasion*.* The incidence of LNM in EGC with submucosal invasion is significantly higher than which with mucosal confined (8–25% vs 2–5%) [5–9].

Nowadays, endoscopic resection (ER) has been widely accepted as an alternative curative procedure for EGC patients without LNM [10, 11]. According to the Japanese gastric cancer treatment guidelines, ER is indicated as a standard treatment for a differentiated-type EGC, of which the depth of invasion is clinically diagnosed as T1a, no ulcerative finding exists, and the tumor size is no more than 2.0 cm [12]. Therefore, it is important to precisely identify those patients with high risk for LNM when deciding the optimal treatment strategy for patients with EGC.

Gastric cancer is the second leading cause of cancer-related death in China, with an estimated more than sixty thousand new cases diagnosed each year [13]. However, the proportion of EGC only accounts about 20% for patients with gastric cancer, and the majority of patients are diagnosed with advanced gastric cancer [13], resulting in a relatively low survival rate of gastric cancer in China compared with Japan and Korea. The present study involved a large number of EGC patients in our institution and retrospectively investigated the incidence of LNM of EGC and the relationship between the clinicopathologic factors and LNM. We aim to identify the risk factors for LNM in EGC patients and provide the basis for further research on individual management of EGC in China.

## Patients and method

### Patients

We consecutively enrolled 1033 patients with EGC who underwent curative gastrectomy with regional lymphadenectomy between 2012 and 2017 at the Department of Gastric Surgery, Fudan University Shanghai Cancer Center, and retrospectively reviewed their medical records. Endoscopic resection was not practiced routinely in our institution, and all the resectable gastric cancer received radical gastrectomy with lymphadenectomy. Patients underwent subtotal or total gastrectomy depending on tumor location, with D1+ or D2 lymphadenectomy in accordance with the guidelines of the Japanese Research Society for Gastric Cancer. We excluded patients with multiple lesions or preoperative treatment history. None of the patients received endoscopic treatment, neoadjuvant chemotherapy, or radiotherapy. Relationships between clinicopathological characteristics and LNM were examined to identify the risk factors. These clinicopathological parameters were analyzed: sex, age, location of tumor, size of tumor, depth of invasion, lymphovascular invasion (LVI), perineural invasion, Lauren classification, and type of differentiation. The ethical committee of Fudan University Shanghai Cancer Center approved this study. The study was performed in accordance with the Declaration of Helsinki and its revisions.

### Histopathologic evaluation

The primary gastric adenocarcinoma and retrieved LN were routinely examined and reviewed by experienced pathologists. Differentiated-type EGC included papillary adenocarcinoma, well or moderately differentiated adenocarcinoma. Poorly or undifferentiated adenocarcinoma and signet ring cell carcinoma were classified as undifferentiated-type EGC. EGC was divided into mucosal and submucosal gastric cancer according to the depth of invasion. Tumor size was recorded as the maximum diameter of the tumor. The analyses of this study were based on postoperative pathology of resected specimens. The pathological staging was performed according to the American Joint Committee on Cancer 7th edition.

### Statistical analysis

The statistical analyses were done using the SPSS software (version 22.0, IBM Inc., Armonk, NY). The risk factors for LNM were identified by performing a Student *t* test, chi-squared test, or Fisher’s exact test. Multivariate logistic regression analysis was used to identify the independent variables associated with LNM. A *P* value of less than 0.05 was considered to be statistically significant.

## Results

### Patient characteristics

A total of 1033 patients with EGC were studied. Of these patients, 668 (64.7%) were men, and 365 (35.3%) were women, ranging in age from 19 to 82 years (mean 56.9 ± 10.9 years). LNM was detected in 173 (16.7%) patients with EGC. Among 508 patients with tumor confined to the mucosa, 44 (8.7%) patients had LNM. In 525 patients with tumor invasion to the submucosa, the incidence of LNM was 24.6% (129 out of 525). One hundred-five (10.2%) of EGC were located in the upper third of the stomach, and 204 (19.7%) were located in the middle, and 724(70.1%) were located in the lower third. The mean diameter of the tumor for EGC was 2.1 ± 1.2 cm. The mean number of retrieved LNs was 24.1 ± 8.2.

### Risk factors for LNM in EGC

The association between LNM and various clinicopathological factors was examined by univariate analysis (Table 1). There were no significant differences between patients with and without LNM in terms of tumor location and number of retrieved LNs. The age, sex, tumor size, invasion depth, lymphovascular invasion (LVI), tumor differentiation, perineural invasion, and Lauren classification showed an association with LNM. Patients with LNM were younger than those without LNM (54.2 ± 11.3 vs 57.4 ± 10.8, *P* < 0.001). The proportion of patients with LNM was higher in female patients than male patients (24.1% vs 12.7%, *P* < 0.001). A greater tumor size (2.5 ± 1.3 cm vs 2.1 ± 1.1, *P* < 0.001) and LVI positive (55.4% vs 12.6%, *P* < 0.001) were related with more frequent LNM. In the patients with undifferentiated-type cancer, the rate of LNM was much higher than the differentiated-type (22.7% vs 8.5%, *P* < 0.001). Perineural invasion was also related to an increased risk of LNM (56.3% vs 15.5%, *P* < 0.001). The diffuse or mixed histologic type showed a more frequent LNM than the intestinal type (*P* < 0.001). Multivariate analysis further showed that age, sex, tumor size, invasion depth, type of differentiation, LVI, and perineural invasion had significant influences on LNM in EGC (Table 2).

**Table 1:** Univariate analysis of risk factors for lymph node metastasis in early gastric cancer

Variables	LN negative (*n* = 860)	LN positive (*n* = 173)	*P*
Age, years	57.4 ± 10.8	54.2 ± 11.3	< 0.001
Sex, *n* (%)			
Male	583 (87.3)	85 (12.7)	< 0.001
Female	277 (75.9)	88 (24.1)	
Location, *n* (%)			
Upper	93 (85.6)	12 (11.4)	0.262
Middle	166 (81.4)	38 (18.6)	
Lower	601 (83.0)	123 (17.0)	
Tumor size, cm	2.1 ± 1.1	2.5 ± 1.3	< 0.001
Retrieved LN	23.9 ± 7.9	25.1 ± 9.3	0.062
Invasion depth			
Mucosal	464 (91.3)	44 (8.7)	< 0.001
Submucosal	396 (75.4)	129 (24.6)	
Lymphovascular invasion, *n* (%)			
Negative	815 (87.4)	117 (12.6)	< 0.001
Positive	45 (44.6)	56 (55.4)	
Perineural invasion, *n* (%)			
Negative	846 (84.5)	155 (15.5)	< 0.001
Positive	14 (43.7)	18 (56.3)	
Differentiation, *n* (%)			
D-type	397 (91.5)	37 (8.5)	< 0.001
UD-type	463 (77.3)	136 (22.7)	
Lauren classification^a^, *n* (%)			
Intestinal	427 (89.9)	48 (10.1)	< 0.001
Diffuse	218 (76.8)	66 (23.2)	
Mixed	127 (74.7)	43 (25.3)	

*D-type* differentiated-type, *LN* lymph node, *UD-type* undifferentiated-type

^a^There are some patients with unknown information about the Lauren classification

**Table 2:** Multivariate analysis of risk factors for lymph node metastasis in early gastric cancer

Variables	Odds ratio	95% confidence interval	*P*
Age	0.976	0.959–0.992	0.004
Male sex	0.47	0.321–0.688	< 0.001
Tumor size	1.272	1.095–1.485	0.002
Submucosal invasion	2.781	1.832–4.221	< 0.001
Undifferentiated-type	2.103	1.362–3.247	0.001
Lymphovascular invasion	5.383	3.318–8.732	< 0.001
Perineural invasion	3.61	1.560–8.306	0.003

### Clinicopathologic characteristics of mucosal cancer compared with submucosal cancer

Table 3 shows the clinicopathologic characteristics of mucosal and submucosal cancers. Age, tumor location, size of the tumor, LNM status, LVI, perineural invasion, and Lauren classification and LN differed significantly between the 2 groups. Patients with submucosal cancer had older age (57.9 ± 10.4 vs 55.8 ± 11.3, *P* = 0.002), a higher proportion of proximal lesion (13.0% vs 7.3%, *P* = 0.011), larger size (2.3 ± 1.1 cm vs 2.0 ± 1.2 cm, *P* < 0.001), more frequent LVI, perineural invasion (16.4% vs 3.0%, *P* < 0.001; 6.1% vs 0.0%, *P* < 0.001, respectively), and more LNM (24.6% vs 8.7%, *P* < 0.001) than patients with mucosal cancer. In terms of Lauren classification, the diffuse or mixed histologic type was observed significantly more often in mucosal cancer than in submucosal cancer (52.9% vs 45.1%, *P* = 0.003).

**Table 3:** Clinicopathologic characteristics of mucosal gastric cancer compared with submucosal gastric cancer

Variables	Mucosal (*n* = 508)	Submucosal (*n* = 525)	*P*
Age, years	55.8 ± 11.3	57.9 ± 10.4	0.002
Sex, *n* (%)			
Male	314 (61.8)	354 (67.4)	0.059
Female	194 (38.2)	171 (32.6)	
Location, *n* (%)			
Upper	37 (7.3)	68 (13.0)	0.011
Middle	104 (20.5)	100 (19.0)	
Lower	367 (72.2)	357 (68.0)	
Tumor size, cm	2.0 ± 1.2	2.3 ± 1.1	< 0.001
Retrieved LN	24.5 ± 8.2	23.7 ± 8.1	0.100
LN metastasis, *n* (%)			
Negative	464 (91.3)	396 (75.4)	< 0.001
Positive	44 (8.7)	129 (24.6)	
Lymphovascular invasion, *n* (%)			
Negative	493 (97.0)	439 (83.6)	< 0.001
Positive	15 (3.0)	86 (16.4)	
Perineural invasion, *n* (%)			
Negative	508 (100.0)	493 (93.9)	< 0.001
Positive	0 (0.0)	32 (6.1)	
Differentiation, *n* (%)			
D-type	217 (42.7)	217 (41.3)	0.653
UD-type	291 (57.3)	308 (58.7)	
Lauren classification^a^, *n* (%)			
Intestinal	213 (47.1)	262 (54.9)	0.003
Diffuse	77 (17.0)	93 (19.5)	
Mixed	162 (35.9)	122 (25.6)	

*D-type* differentiated-type, *LN* lymph node, *UD-type* undifferentiated-type

^a^There are some patients with unknown information about the Lauren classification

### Risk factors for LNM in mucosal gastric cancer and submucosal gastric cancer

Univariate analysis indicated that age, sex, tumor size, LVI, differentiation type, and Lauren classification were risk factors for LNM (Table 4). The multivariate analysis showed that female sex, large tumor size, undifferentiated-type, and LVI were independent risk factors for LNM (Table 5). Univariate analysis indicated that age, sex, tumor size, LVI, perineural invasion, differentiation-type, and Lauren classification showed an association with LNM in patients with submucosal gastric cancer (Table 4). The multivariate analysis indicated that female sex, younger age, LVI, and perineural invasion were the independent risk factors for LNM (Table 6). Female sex and LVI were both independent risk factors for LNM in mucosal and submucosal gastric cancer.

**Table 4:** Risk factors for lymph node metastasis in mucosal and submucosal gastric cancer

Variables	Mucosal gastric cancer			Submucosal gastric cancer		
	LN (−) (*N* = 464)	LN (+) (*N* = 44)	*P*	LN (−) (*N* = 396)	LN (+) (*N* = 129)	*P*
Age, years	56.4 ± 11.2	49.8 ± 11.6	< 0.001	58.7 ± 10.2	55.7 ± 10.8	0.005
Sex, *n* (%)			< 0.001			0.001
Male	301 (95.9)	13 (4.1)		282 (79.7)	72 (20.3)	
Female	163 (84.0)	31 (16.0)		114 (66.7)	57 (33.3)	
Location, *n* (%)			0.25			0.226
Upper	36 (97.3)	1 (2.7)		57 (83.8)	11 (16.2)	
Middle	92 (88.5)	12 (11.5)		74 (84.0)	26 (26.0)	
Lower	336 (91.6)	31 (8.4)		265 (74.2)	92 (25.8)	
Tumor size, cm	1.9 ± 1.1	2.6 ± 1.4	< 0.001	2.2 ± 1.1	2.5 ± 1.2	0.007
Retrieved LN	24.3 ± 8.1	26.3 ± 9.0	0.126	23.3 ± 7.6	24.7 ± 9.5	0.085
Lymphovascular invasion, *n* (%)			< 0.001			< 0.001
Negative	456 (92.5)	37 (7.5)		359 (81.8)	80 (18.2)	
Positive	8 (53.3)	7 (46.7)		37 (43.0)	49 (57.0)	
Perineural invasion, *n* (%)			NA			< 0.001
Negative	464 (91.3)	44 (8.7)		382 (77.5)	111 (22.5)	
Positive	0 (0.0)	0 (0.0)		14 (43.8)	18 (56.2)	
Differentiation, *n* (%)						< 0.001
D-type	214 (98.6)	3 (1.4)	< 0.001	183 (84.3)	34 (15.7)	
UD-type	250 (85.9)	41 (14.1)		213 (69.2)	95 (30.8)	
Lauren classification^a^, *n* (%)			< 0.001			< 0.001
Intestinal	207 (97.1)	6 (2.9)		220 (84.0)	42 (16.0)	
Diffuse	139 (85.7)	23 (14.3)		79 (64.8)	43 (35.2)	
Mixed	68 (88.3)	9 (11.7)		59 (63.4)	34 (36.6)	

*D-type* differentiated-type, *LN* lymph node, *NA* not available, *UD-type* undifferentiated-type

^a^There are some patients with unknown information about the Lauren classification

**Table 5:** Multivariate analysis of risk factors for lymph node metastasis in mucosal gastric cancer

Variables	Odds ratio	95% confidence interval	*P*
Age (> 60)	0.981	0.953–1.01	0.205
Male sex	0.341	0.163–0.715	0.004
Tumor size (> 2 cm)	1.38	1.07–1.779	0.013
Undifferentiated-type	6.873	1.964–24.05	0.003
Lymphovascular invasion	11.156	3.088–40.307	< 0.001

**Table 6:** Multivariate analysis of risk factors for lymph node metastasis in submucosal gastric cancer

Variables	Odds ratio	95% confidence interval	*P*
Age (> 60)	0.976	0.956–0.997	0.026
Male sex	0.557	0.354–0.875	0.011
Tumor size (> 2 cm)	1.2	0.993–1.45	0.059
Undifferentiated-type	1.582	0.976–2.565	0.062
Lymphovascular invasion	4.891	2.907–8.229	< 0.001
Perineural invasion	3.673	1.62–8.325	0.002

## Discussion

EGC is more commonly diagnosed in Japan, where EGC accounts approximately 60% for all patients diagnosed as gastric cancer [2]. By contrast, the proportion of EGC in China varies from 10% to 20%. Such great discrepancy might be partly but not fully interpreted by the fact of more screening programs and different interpretations of pre-cancerous lesions in Japan, which furtherly has a large influence on the results of those studies analyzing risk factors for LNM in EGC. In Japan, regardless of the presence of invasive findings, not only structural atypia but also cellular atypia is sufficient to make a diagnosis of gastric cancer [14, 15]. However, these lesions could be diagnosed instead as high-grade dysplasia in our institution. This difference might result in a relatively higher incidence of EGC, a corresponding superior prognosis, and a lower incidence of LNM in patients with EGC in the Japanese cohort. Another reason might be the different efforts in pathological evaluation for early gastric cancer. For minority cases with conventional HE diagnosis of lymph node metastasis, immunohistochemistry will be added for further diagnosis. The thickness of paraffin sections is generally 4–6 μm. Thin slice specimen is not a routine in our center. Thus, the pathological staging of gastric cancer may be underestimated, resulting in a high incidence of lymph node metastasis rate. In addition, the worldwidely accepted indications of ER for EGC are mainly based on the clinical trials for Japanese patients and might not be exactly applicable to the patients in our country. It is important to define those patients benefiting from ER without compromising long-term survival or those patients being at high risk of LNM to receive curative gastrectomy instead of ER. Thus, the present study enrolled a relatively large number of EGC patients in a single institution from China and retrospectively investigated the incidence and risk factors for LNM in a Chinese population.

The status of LNM is the major concern in deciding the optimal treatment modality for EGC [16, 17]. In the present study, LNM occurred in 16.7% of all EGC patients, with 8.7% in mucosal and 24.6% in submucosal gastric cancer, respectively. Our study found a relatively high LNM incidence in patients with EGC, which is notably higher than that of the Japanese cohort [2, 18]. Despite the different rates of LNM, analyses of risk factors for LNM revealed a similar finding with those reported in the literature. Several studies had suggested large tumor size, ulcerative findings, submucosal invasion, LVI, and undifferentiated-type cancer to be risk factors for LNM in EGC and recommended gastrectomy with LN dissection as the treatment option for those patients at high risk of LNM [19–21]. The present study also indicated that age, sex, tumor size, invasion depth, type of differentiation, LVI, and perineural invasion had significant effects on LNM in EGC by univariate and multivariate analyses. Depth of invasion is one of the most important risk factors for LNM. In our study, we compared clinicopathologic characteristics of mucosal cancer with submucosal cancer and did a subgroup analysis of risk factors in the mucosal cancer group and submucosal cancer group, respectively. Patients with submucosal gastric cancer had an older age, a higher proportion of upper lesion, larger tumor size, more frequent lymphovascular invasion and perineural invasion, and more LNM than patients with mucosal gastric cancer. Female sex and LVI were found to be the common risk factors in the two subgroups.

Majority of studies reported that more aggressive biological behavior and higher risk of metastasis were more frequently showed in young gastric cancer patients. Takatsu et al. [22] reported that younger patients are more likely to present with LNM, indicating that age was an independent risk factor. Another study by Lee et al. [23] reported that old age was associated with a lower risk for LNM in patients with EGC. This might result partly from a lower proportion of differentiated-type cancer in younger patients with EGC. However, other studies reported that there was no significant difference in LNM incidence or overall survival between younger and older patients [24]. In the present study, young age was demonstrated to be an independent risk factor for LNM in EGC patients. In the subgroup analysis, age also had an independent association with LNM in patients with submucosal gastric cancer.

Our study also indicated that female sex was an independent risk factor for LNM in EGC patients. The incidence of LNM was statistically higher in female patients than male patients. Other researchers also have reported that female sex is associated with LNM in EGC [25, 26]. In female patients, the biological behavior of gastric cancer might be more aggressive, which could not be fully defined by tumor size, depth of invasion, or LVI [27]. Further studies were needed to investigate the biological association between sex and LNM.

In agreement with previously reported studies, our study also demonstrated that the tumor size, depth of invasion, and LVI are statistically significant risk factors for LNM in EGC. A previous study reported that no LNM was detected in mucosal cancers less than 2 cm, indicating tumor size as an independent risk factor for LNM [28]. Generally, the depth of tumor invasion is tightly related with LNM in EGC. Both univariate and multivariate analyses in the present study demonstrated that a larger size tumor, submucosal invasion, and the presence of LVI and perineural invasion were significant predictive factors for LNM in patients with EGC. Among these risk factors, LVI might be the most important risk factor for LNM. In the present study, LVI was associated with LNM in both mucosal and submucosal lesions. Therefore, EGC with LVI is associated with a higher frequency of LNM, and surgical resection is necessary for EGC patients at high risk of LNM.

In our study, the rate of LNM in undifferentiated-type EGC was obviously higher than in differentiated-type. Undifferentiated-type was an independent risk factor for LNM in EGC. The more aggressive features of undifferentiated-type than the differentiated-type, have already been demonstrated [29]. Although ER has been indicated for undifferentiated EGC according to the expanded criteria, ER has generally been limited to differentiated-type EGC because of the higher risk of LNM in undifferentiated EGC. In the subgroup analyses, the present study also confirmed that undifferentiated-type was an independent risk factor for LNM in mucosal cancer, whereas it was not in submucosal gastric cancer. Among these risk factors, LVI might be a more important factor for LNM in submucosal gastric cancer. In the present study, LVI was significantly associated with LNM in both mucosal and submucosal EGC.

We would indicate the criteria for endoscopic resection as those male patients with a differentiated-type EGC, of which the depth of invasion is clinically diagnosed as T1a, no ulcerative finding exists, no lymphovascular invasion, and the tumor size is no more than 2.0 cm. Of 157 patients who met the above criteria in our study, no one has lymph node metastasis. Endoscopic resection should be considered for tumors that have a very low possibility of lymph node metastasis and are suitable for en bloc resection. According to the Japanese guidelines, tumors of the following categories have a very low possibility of lymph node metastasis when they are not accompanied with lymphovascular infiltration and could be indicated for endoscopic resection (expanded indication): of differentiated-type, ulcer (−), but > 2 cm in diameter; of differentiated-type, ulcer (+), and < 3 in diameter; and of undifferentiated-type, ulcer (−), and < 2 cm in diameter. However, our research indicated that submucosal gastric cancer has a high incidence of lymph node metastasis. We do not recommend ER for submucosal gastric cancer. Nowadays, different gastric cancer treatment guidelines have different indications for endoscopic treatment of early gastric cancer. But the general principle should be the same, and only patients with a very low risk of lymph node metastasis are suitable for endoscopic treatment. Patients at high risk for lymph node metastasis should still undergo standard surgical resection and lymph node dissection. Our results show that the rate of lymph node metastasis in early gastric cancer in China is relatively high. If the endoscopic treatment guideline of Japanese and Korean is completely applied, a large part of patients with a higher risk of lymph node metastasis would receive endoscopic resection. Endoscopic treatment alone cannot achieve a curative resection for those patients, greatly increasing the possibility of recurrence and distant metastasis after endoscopic resection. Therefore, we must carefully choose the indications for endoscopic treatment. We cannot arbitrarily expand the indications for endoscopic treatment and blindly exaggerate the effect of endoscopic treatment. The overall long-term survival rate of patients remains the main therapeutic goal pursued.

Although the present study is one of the largest retrospective studies for EGC from a single institution in China, there are still some limitations. First, it was a retrospective study based on medical records in a single center. Second, only patients who underwent surgery were included in this study. Thus, possible selection bias was unavoidable. Third, our results are based on pathologic findings and not on clinical and endoscopic findings. Moreover, the depth of submucosal invasion is not specified (SM1, SM2, or SM3) in the present study. Therefore, our study might be not powerful enough to confirm all these related issues. To successfully utilize the endoscopic resection in China, criteria need to be strictly applied to the appropriate population, and further research including prospective, randomized, and controlled studies should be conducted.

## Conclusions

In conclusion, our study revealed a relatively high incidence of LNM in Chinese patients with EGC. Sex, age, tumor size, depth of invasion, differentiation type, and lymphovascular and perineural invasion were identified as independent risk factors for LNM in EGC. LNM should be considered when deciding on the management of EGC. Minimal invasive treatment, such as ER, may be acceptable as a curative treatment in highly selective patients. Radical gastrectomy with LN dissection should be performed in EGC patients with a high risk of LNM in a Chinese population.

## Data Availability

The datasets generated and/or analyzed during the current study are not publicly available because they are derived from the patient database of the center and hence subject to confidentiality but are available from the corresponding author on reasonable request.
